# BAT6026, a novel anti-OX40 antibody with enhanced antibody dependent cellular cytotoxicity effect for cancer immunotherapy

**DOI:** 10.3389/fonc.2023.1211759

**Published:** 2023-07-28

**Authors:** Shizhong Liang, Dandan Zheng, Xiong Liu, Xiong Mei, Congcong Zhou, Cuizhen Xiao, Chao Qin, Haitao Yue, Jian Lin, Cuihua Liu, Shengfeng Li, Jin-Chen Yu

**Affiliations:** ^1^ Department of Discovery Research, Bio-Thera Solutions, Ltd., Guangzhou, Guangdong, China; ^2^ Department of Technology Development, Bio-Thera Solutions, Ltd., Guangzhou, Guangdong, China

**Keywords:** OX40, antibody, ADCC-enhanced, immune checkpoint, cancer immunotherapy, BAT6026

## Abstract

OX40 (CD134), a member of the TNF receptor superfamily, is a widely studied costimulatory immune checkpoint. Several OX40 agonistic antibodies are in the clinical stage for cancer treatment, among which PF-04518600 is the leader and currently in phase II trial. It has been recognized that one potential mode of action for anti-OX40 antibodies is the deletion of intratumoral Tregs. Thus, a novel human anti-OX40 antibody, BAT6026, was generated with enhanced antibody dependent cellular cytotoxicity (ADCC) *via* fucose deletion to strengthen its Treg depletion activity. This characteristic of BAT6026 differentiates it from other previously reported anti-OX40 antibodies in the field of tumor therapy. The affinity of BT6026 to OX40 was 0.28nM, approximately 8 times stronger than that of PF-04518600. BAT6026 effectively competed for the binding of ligand OX40L to OX40, whereas PF-04518600 only partially competed. Moreover, compared to PF-04518600, BAT6026 activated T cells more effectively when clustered by FcγRs engagement and stimulated SEB-pretreated PBMCs to secrete IL-2 cytokines *in vitro*. In addition, BAT6026 demonstrated stronger anti-tumor activity than PF-04518600 in an OX40-humanized mouse MC38 tumor model. BAT6026 also showed a significantly synergistic effect on tumor inhibition when combined treatment with PD-1 antibody. Analysis of tumor-infiltrating T cells revealed that BAT6026 treatment significantly reduced Treg cells and increased CD8+ T cells in tumor. Preclinical safety assessment in non-human primates demonstrated a good safety profile for BAT6026. Together these data warrant further development of BAT6026 into clinical trials for patients with cancer.

## Introduction

In recent years, the landscape of cancer treatment has been altered by the advent of immunotherapy, which has offered improved survival in several solid cancers and established itself as a new therapeutic modality. Among these immunotherapies, Ipilimumab, an antibody targeting CTLA-4, was the first approved checkpoint inhibitor. PD-1 and PDL-1 antibodies, blocking PD-1 pathway and thus activating T cells, are the second wave receiving regulatory approval (1). Recently, Relatlimab, an anti- LAG3 antibody, was approved for the treatment of unresectable or metastatic melanoma by FDA in combination with anti-PD-1 Nivolumab (2). Although these immunotherapies provide a new direction for cancer treatment, their overall response rates (ORR) as single treatment were generally only ~20% (3). Hence, the development of other novel immunotherapies to enhance clinical effectiveness is required (4). Since the approved anti-checkpoint antibodies all belong to inhibitory immune checkpoints, now targeting on stimulatory immune checkpoints may have a better chance of success as they also share responsibility in regulating immune cells (5, 6).

Many immunostimulatory checkpoints belong to the tumor necrosis factor receptor superfamily (TNFRSF), such as OX40, GITR, 4-1BB, CD40, and CD27 (7). OX40 (CD134) is a type I transmembrane glycoprotein composed of 275 amino acids and mainly expressed on activated immune cells, primarily CD4+ T cells, CD8+ T cells and intratumoral Tregs (8). Its only known ligand, OX40L (CD252), is a type II transmembrane glycoprotein and expressed mainly on activated antigen-presenting cells induced by CD40, toll-like receptors, and inflammatory cytokines (9, 10). When binding to one OX40L trimer, three OX40 molecules are clustered, which directly activate NF-κB signal pathway as well as augment PI3K/PKB and NFAT pathways of the T cell receptor (TCR) (11, 12). These signal pathways account for the functional consequences of the division, survival and cytokine secretion of T cells. Although the cellular mechanism of OX40 antibody underlying anti-tumor immunity is not completely clear, its action has been widely recognized to contain three potential modes: (1) directly stimulating CD4+ and CD8+ T cells by enhancing their proliferation and survival; (2) inhibiting Tregs by reducing their suppressive function; (3) directly deleting intratumoral Tregs by engaging Fcγ receptors on effector cells (13, 14).

In mouse tumor models, previous agonistic OX40 antibodies have shown remarkable anti-tumor efficacy as a single treatment or in combination with other immunotherapies (5). The antitumor efficacy was further reported to be mainly caused by the depletion of intratumoral regulatory T cells *via* ADCC effect (15, 16). In addition, OX40 was found to be expressed at high level on tumor Treg cells but at low level on tumor Teff cells in many types of human tumor (17–19). These data suggest that strengthening Treg depletion function of OX40 antibody may largely improve its antitumor activity in cancer patients. Besides Treg depletion mechanism, the agonistic anti-OX40 can also inhibit tumor by augmenting activation and proliferation of CD4 +T cells that results into activation of CD8 +T cells (20, 21).

To date, there are several anti-OX40 antibodies in clinical stage for cancer indication, including PF-04518600 (Pfizer), BMS-986178 and GSK3174998. The former two are in phase II and the latter two are in phase I trials, yet none of them is ADCC-enhanced (22). Considering the important role of Treg depletion in the anti-tumor effect of OX40 antibodies, we hypothesized that ADCC-enhanced OX40 antibodies may have stronger clinical efficacy than non-ADCC-enhanced OX40 antibodies. Herein we developed a novel ADCC-enhanced anti-OX40 antibody aiming to strengthen its Treg depletion activity, BAT6026. BAT6026 demonstrated favorable *in vitro* characteristics, mouse tumor model efficacy, and a good safety profile in monkey toxicity study. It is currently being tested in phase I trial.

## Materials and methods

### Cell lines and reagents

Raji cells were purchased from the National Collection of Authenticated Cell Culture. Jurkat cells stably expressing human OX40 were prepared in house. Jurkat cells stably expressing human OX40 in pCMV vector (SinoBiological) and NF-κB-luciferase construct in pGL4.32 vector (Promega) were prepared in house. Jurkat cells stably expressing human FcγRIIIa (158V) in pCMV cector (SinoBiological) and NFAT-luciferase construct in pGL4.32 vector (Promega) were prepared in house. Recombinant extracellular domains (ECD) of OX40 from different species and OX40L-mFC (human OX40 ligand fused with mouse Fc domain) were purchased from ACRO. Recombinant human CD27, CTLA-4, GITR, CD40 and PD-1 were also purchased from ACRO. Goat anti-human kappa light chains-HRP secondary antibody was purchased from Sigma-Aldrich. ONE-Glo™ Luciferase Assay System and CytoTox 96^®^ Non-Radioactive Cytotoxicity Assay kit were purchased from Promega. Human peripheral blood mononuclear cells (PBMCs) were purchased from Leide Bioscience. Staphylococcus enterotoxin B (SEB) was purchased from Invitrogen. The IL-2 detection kit was purchased from Mabtech.

### Antibody screening, optimization and generation

Using the method described in the literature of Michael et al, a yeast display library of completely human antibodies was constructed (23). First, the DNA fragments of the heavy chain variable region (VH) and the light chain variable region (VL) of human IgM and IgG gene were obtained by PCR technique. These VH and VL fragments were then assembled into scfvs *via* overlapping PCR reactions. The scfvs were inserted into the yeast display plasmid PYD1. Finally, a large number of these PYD1 plasmids were transduced into Saccharomyces cerevisiae by electroporation to obtain a yeast display library with an approximate size of 5×10^9^.

To obtain candidate antibodies specifically targeting OX40, the yeast antibody library was screened and enriched using OX40-coupled magnetic beads and fluorescence activated cell sorting (FACS). After initial screening and cell-based binding analysis, the positive candidates were subjected to further examination in cell-based function assays and animal efficacy studies to select the clinical candidates. The affinity of the clinical candidate was further improved by applying “DNA walking” technique (24).

BAT6026 was expressed in a FUT8 (alpha-(1,6)-fucosyltransferase)-knockout CHO cell line established in house. BAT6026-wt is a regularly fucosylated form of BAT6026, and was expressed in wild type CHO cell. PF-04518600 was prepared in house using heavy and light chain sequences from a patent (US 9,028,824 B2). BAT6026-mIgG2a is a fusion antibody with Fab domain of BAT6026 and Fc domain of mouse IgG2a lacking fucose modification, while BAT6026-mIgG2a-wt has the Fc domain of mouse IgG2a regularly fucosylated. PF-04518600 and BAT6026-mIgG2a-wt were transiently expressed in HEK293 cells, and BAT6026-mIgG2a was transiently expressed in a FUT8-knockout CHO cell line.

### Generation of the Fut8-knockout CHO cell line

The Fut8-knockout CHO cell line was established using TALEN gene editing technology, described in patent ZL201810910890. Firstly, the genome sequence of Fut8 (Gene ID: 100751648) was obtained by analyzing the complete genome sequence of Chinese hamster ovary cells, CHO-K1 (NW-003613860). Because the activity center of FUT8 enzyme resides in the region encoded by exon 1, the left and right flank sequences of exon 1 were chosen as the targeting sequences of TALEN technology. The Fut8-targeting TALEN plasmids were constructed and transfected into CHO-K1 cells using Lipofectamine 2000 reagent. Subsequently, the positive candidate clones with inactivated FUT8 enzyme were obtained by flow cytometry screening of cell surface characteristics. Genome sequencing was used to confirm the homozygous knockout of the Fut8 gene in the selected CHO cell line.

### OX40 specific binding assay

Microtiter plates were coated with the ECD of human OX40, cynomolgus OX40, mouse OX40, rat OX40, human CD27, CTLA-4, GITR, CD40 or PD-1 at 4°C overnight. After washing, the coated antigens were incubated with serial dilutions of BAT6026 for 1 h at 37°C. Goat anti-human kappa light chains-HRP secondary antibody was added to wells after washing and incubated for 30 min at 37°C. Color appeared after HRP substrate TMB was added to the wells, and the plates were read on the SpectraMax (Molecular Devices) at 450 nm.

### Binding to target-expressed cells

Jurkat cells stably expressing human OX40 were incubated with serial dilutions of BAT6026 or PF-04518600 antibody for 60 min in PBS with 1% BSA at 4°C, followed by washing and subsequent incubation with PE-labeled goat anti-human Fc secondary antibody for 25min at 4°C. Then cells were washed and resuspended in PBS with 1% BSA at 4°C, followed by flow cytometry analysis and calculation of mean fluorescence intensity (MFI) performed on an Accuri C6 system (BD Biosciences).

### Affinity measurement

Surface plasmon resonance (SPR) analysis was carried out using Protein A sensor chips (GE Healthcare) for measuring affinity kinetics between antibodies and OX40 antigen. Antibodies were diluted with HBS EP+ running buffer to 5 µg/ml and were first immobilized onto the sample flow cell of Protein A sensor chip with a flow rate of 10 μl/min at 25°C, and the reference flow cell was left blank. OX40 antigens were serially diluted with HBS EP+ running buffer, then injected over the two flow cells at a range of eight concentrations using a single-cycle kinetics program. HBS EP+ running buffer was also injected using the same program for background subtraction. All data were fitted to a 1:1 binding model using Biacore T200 Evaluation Software 3.1.

### OX40L blocking assay

Jurkat cells stably expressing human OX40 were incubated with OX40L-mFC and serial dilutions of BAT6026 or PF-04518600 antibody for 1h in PBS containing 1% BSA at 4 °C. After cells were washed, the bound OX40L-mFc was detected by incubation with PE-labeled goat anti-mouse Fc secondary antibody. Then cells were washed and resuspended in PBS with 1% BSA at 4 °C, followed by flow cytometry analysis and calculation of MFI performed on an Accuri C6 system.

### T cell activation measured by a luciferase reporter assay

Jurkat cells stably expressing human OX40 and NF-κB-luciferase construct and equal number of Raji cells were mixed and co-incubated with serial dilutions of BAT6026, PF-04518600 antibody or an control IgG1 for 5h at 37°C. Then the luciferase activity of samples were determined by a SpectraMax reader following the user guide of the ONE-Glo™ Luciferase Assay System (Promega).

### PBMCs activation assay

2×10^5^ PBMCs were added into each well of 96-well microtiter plates and pre-incubated with 90ng/mL SEB for 24h at 37°C. Then serial dilutions of BAT6026, PF-04518600 antibody or an isotype-matched negative control human IgG1 were added. After incubation at 37°C for 4 days, IL-2 secreted from activated PBMCs was determined by using an IL-2 detection kit (Mabtech).

### ADCC assay with luciferase reporter

Jurkat cells stably expressing human FcγRIIIa (158V) and NFAT-luciferase construct were used as effector cells, and Jurkat cells stably expressing human OX40 were used as target cells. The effector-to-target cell ratio in the assay was 2.5:1. Serial dilutions of BAT6026 or BAT6026-wt antibody were added to the cells and incubated for 4 hrs at 37°C. Then the luciferase of samples were determined by a SpectraMax reader following the user guide of the ONE-Glo™ Luciferase Assay System (Promega).

### ADCC assay with PBMCs

SEB-activated human PBMCs from healthy donors were used as effector cells and Jurkat cells stably expressing human OX40 were used as target cells. The effector-to-target cell ratio in the assay was 25:1. The cell ratios in this and above ADCC assays were optimized using various effector-to-target cell ratios, and selected based on good assay reproducibility and large signal-to-noise window. Serial dilutions of BAT6026 or BAT6026-wt antibodies were added to the cells, and incubated for 4 hrs at 37°C in RPMI 1640 with 10%FBS. Released lactate dehydrogenase (LDH) in culture supernatants was measured using a SpectraMax reader and CytoTox 96^®^ Non-Radioactive Cytotoxicity Assay kit (Promega), according to the manufacturer’s instructions.

### MC38 tumor model in OX40-humanized and PD-1/OX40-dual-humanized mice

1×10^6^ MC38 cells were implanted subcutaneously into the right flank of OX40-humanized or PD-1/OX40-humanized female mice, whose ECD of OX40 or PD-1/OX40 was replaced by human counterpart (Biocytogen). When the tumor volume reached approximately 100 mm^3^, mice were randomly allocated into each study group and intraperitoneally injected with test antibodies once every 3 days for a total of 6 times. Tumor volume and body weight were measured twice a week, and mice were euthanized when the tumor volume reached 3000 mm^3^, or the percentage of body weight loss exceeded 20%.

### Analysis of tumor infiltrating lymphocytes and splenocytes with flow cytometry

OX40-humanized mice were implanted with MC38 tumor cells and treated with OX40 antibodies on days 9 and 14 post implantation. On day 17, tumors were collected and dissociated into single cell suspensions by using a digestive solution (1640 medium + 2%FBS + Collagenase IV (Sigma, C5138) + DNase I (Sigma, D5025)), and spleens were ground with sterilized glass slides and filtered through a steel mesh. Red blood cells were lysed using red cell lysing buffer (TIANGEN, RT122). Single cell suspensions were first incubated with the LIVE/DEAD™ Fixable Green Dead Cell Stain Kit (Invitrogen, L34970), then labeled with the following antibodies in flow cytometric analyses: Brilliant Violet 421™ anti-mouse CD3 (BioLegend, 100228), PE anti-mouse Ki-67 (BioLegend, 652404), PerCP anti-mouse CD8a (BioLegend, 100732), APC/Cyanine7 anti-mouse CD45 (BioLegend, 103116), PE/Cyanine7 anti-mouse IFN-γ (BioLegend, 505826), eFluor™ 450 anti-FoxP3 (Invitrogen, 48-5773-82) and eFluor™ 506 anti-CD4 (Invitrogen, 69-0042-82). Intracellular FoxP3, IFN-γ and Ki67 were labeled following the product manual. Flow cytometry analysis was performed using Cytek^®^ Aurora (Cytek Biosciences).

### GLP toxicity study of BAT6026 in cynomolgus monkeys

In the repeated-dose toxicology study, cynomolgus monkeys were injected intravenously with BAT6026 at doses of 1, 5 and 30 mg/kg (5 male and 5 female monkeys in each group) once per week (QW), for a total of 5 doses, followed by a 4-week recovery period. After the 5th dose, 3 monkeys per gender/group were euthanized and autopsied, and the remaining 2 monkeys in each gender/group were observed for an additional 28 days prior to being euthanized. The following parameters were examined during the study: clinical observations, ophthalmology, food consumption, body weight, body temperature, clinical pathology, lymphocyte subpopulation, immunoglobulins, toxicokinetics, immunogenicity, local skin reactions at the injection site, safety pharmacology, organ weight and ratios, gross pathology and histopathology.

### Statistical analysis

All statistical analyses were performed using GraphPad Prism (version 8). Statistical significance between groups was determined using t-test or one-way ANOVA. A p value of ≤ 0.05 was considered statistically significant (*p ≤ 0.05, **p ≤ 0.01,***p ≤ 0.001, and ****p ≤ 0.0001).

## Result

### Generation and affinity determination of BAT6026

A yeast display library of full human antibodies was constructed by cloning the DNA fragments of the VH and VL from human IgM and IgG gene in the form of scFv structure (23). Using human OX40-coupled magnetic beads and fluorescence activated cell sorting to screen the human antibody library, several candidate antibodies specifically targeting OX40 were obtained (25). These candidates were further selected using purified antigen and cell-based binding assays, cell-based function assays and animal efficacy studies, as well as through process of affinity maturation using “DNA walking” technique (24), to achieve the clinical candidate BAT6026.

**Figure 1:**
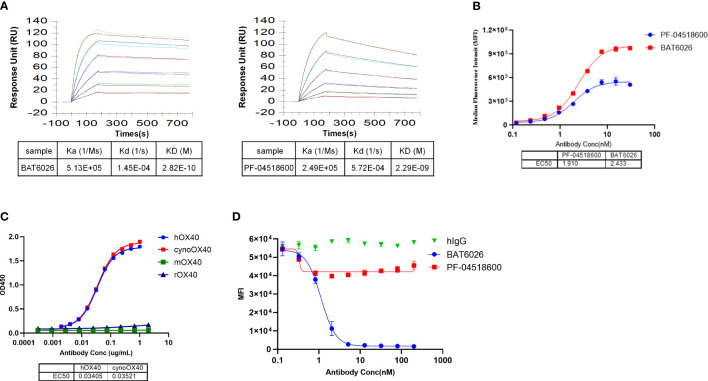
Affinity determination, binding selectivity and ligand blocking of BAT6026 to OX40. **(A)** Dynamic Curves of BAT6026 and PF-04518600 blinding to human OX40 (His Tag) was detected using BIAcore T200 in 1:1 binding model. BAT6026 or PF-04518600 was set as ligands immobilized on CM5 chips. Human OX40 in different concentrations were set as analytes. **(B)** Applying cell-based binding assay and FACS to profile the affinity of BAT6026 or PF-04518600 to human OX40 expressed on Jurkat cells. **(C)** Using ELISA to compare the affinity of BAT6026 to extracellular domains of human OX40 (hOX40), monkey OX40 (cynoOX40), mouse OX40 (mOX40) or rat OX40 (rOX40). **(D)** Cell-based ligand competition assay. Increasing amounts of BAT6026 or PF-04518600 were incubated with Jurkat cells overexpressing human OX40 in the presence of 50ug/ml OX40L. The cell-bound OX40L was subsequently detected using Streptavidin R-Phycoerythrin Conjugate.

Many characterization assays of BAT6026 were performed, including PF-04518600 as a comparator since it is the currently leading antibody in the anti-OX40 field (26). The binding affinity of BAT6026 to purified human OX40 antigen was evaluated utilizing SPR technology (BIAcore 2000). As shown in **Figure 1A**, the equilibrium dissociation constant (KD value) of BAT6026 to OX40 was 0.282nM, approximately 8-fold stronger than that of PF-04518600. The ability of BAT6026 to bind to OX40 on cells was measured using Jurkat cells expressing human OX40. BAT6026 bound OX40 expressed on Jurkat cells with an EC50 value of 1.91nM, similar to that of PF-04518600. However, the maximal binding signal value of BAT6026 was about twice higher than that of PF-04518600 (**Figure 1B**).

### Binding selectivity and ligand blocking of BAT6026 to OX40

To study binding specificity, the affinity of BAT6026 binding to human, cynomolgus, mouse and rat OX40 were examined by ELISA. These OX40 antigens were complete ECD of OX40s of each species. As shown in **Figure 1C**, BAT6026 had a high and similar affinity to human and monkey OX40 with an EC50 value of 0.034ug/ml and 0.035ug/ml, respectively. In contrast, BAT6026 did not bind to ECD of mouse or rat OX40 (**Figure 1C**). The binding specificity of BAT6026 against other immune checkpoint proteins was further examined. Results demonstrated that BAT6026 did not bind to ECD of CD27, CTLA-4, GITR, CD40 and PD-1 at all (data not shown).

Next, we tested whether BAT6026 could block the binding of OX40L to OX40, using jurkat cells overexpressing OX40. As shown in **Figure 1D**, BAT6026 significantly blocked the binding of OX40L to OX40 on the cell surface with an IC50 of 1.124nM, while PF-04518600 only moderately blocked. Since the binding affinities of BAT6026 and PF-04518600 to OX40 on the cell surface are similar, this difference in OX40L blocking is likely due to different binding regions of OX40 they recognize.

### Biological effect of BAT6026 on T cell activation *in vitro*

**Figure 2:**
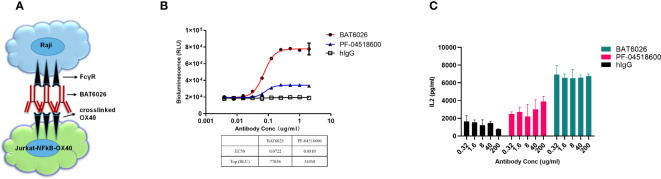
Biological effect of BAT6026 on T cell activation measured by cell-based function assays. **(A)** Schematic diagram of the established cell function assay based on NF-kB luciferase reporter assay. Various concentrations of anti-OX40 antibodies were co-incubated with Raji cells and Jurkat T cells overexpressing human OX40 and NFkB-driven luciferase for 6 hours. The induced luciferase activity in Jurkat cells was measured using a luciferase detection kit. **(B)** Activation function of BAT6026 or PF-04518600 on T cell was detected using the NF-kB luciferase reporter assay. **(C)** PBMCs from healthy donors were pre-activated by super antigen SEB (Staphylococcal enterotoxins B) (40ug/ml) and then co-incubated with BAT6026 or PF-04518600 for four days. The secreted IL-2 was then measured by ELISA.

Clustered OX40 can stimulate T cells and strengthen the survival of T cells through pathways including NF-kB signal (13). Therefore, an NF-kB luciferase reporter assay was developed to determine the effect of BAT6026 on T cell activation. This reporter assay included Jurkat cells overexpressing human OX40 and NFkB-driven luciferase, and Raji cells expressing endogenous FcγRs (**Figure 2A**). As shown in **Figure 2B**, following binding to OX40 on T cell and subsequent crosslink offered by FcγRs on the Raji cells surface, BAT6026 demonstrated a dose-dependent fashion on T cell activation, with an EC50 of 0.0722 ug/ml. In this study, PF-04518600 exhibited a comparable EC50 in the study, however the maximal luciferase activity it provoked was much lower than that induced by BAT6026. This is consistent with the lower maximal binding signal value of PF-04518600 than that of BAT6026 (**Figure 1B**).

To further assess the *in vitro* functional activity of BAT6026 on T cells, we measured its ability to provoke IL-2 cytokines release from SEB-pretreated PBMCs. In this assay, all concentrations (0.32-200 µg/ml) of BAT6026 induced approximately 4-fold IL-2 secretion over the background level of IgG control (**Figure 2C**). Although PF-04518600 appeared to show a minor dose-dependent increase of IL-2 release at these concentrations, it induced significantly lower IL-2 release than BAT6026, even at the highest concentration.

The mechanism of action of BAT6026 is to strengthen the immune function by activating T cells. Such type of agonistic antibody may over-activate the immune system and cause severe cytokine storms (27, 28). To examine whether BAT6026 may cause this harm, its ability to provoke cytokines release from unactivated PBMCs was measured. As shown in **Figure S1**, BAT6026 did not stimulate unactivated PBMCs to produce IL-2, IFN-γ, IL-6, and TNF-α even at concentration up to 200ug/mL, compared to the positive control anti-CD28 antibody which caused thousands-fold more cytokines release at 200ug/ml. The data implicated a high safety margin of BAT6026 in clinical trials.

### Enhanced ADCC of BAT6026 *in vitro*

Considering that one potential mode of action for OX40 antibodies is directly deleting intratumoral OX40-expressed Tregs (17–19), we hypothesized that it might be beneficial in clinical trials to enhance ADCC effect on OX40 antibody. Therefore, BAT6026 was expressed in a Fut8- knockout CHO cell line. Antibody expressed in this cell line was completely devoid of fucose modification (**Supplementary Table 1**), thus harboring a strengthened ADCC activity (29, 30). BAT6026 expression plasmid was also transfected into a wt CHO cells, and the product with normal level of fucose modification and ADCC activity was designated as BAT6026-wt for comparison. As the gamma Fc receptor IIIa (Fcγ-RIIIa) is the major Fc receptor mediating ADCC effect (31), we first used SPR technology to evaluate the affinity of BAT6026 and BAT6026-wt to Fcγ-RIIIa. **Table 1** shows that afucosylated BAT6026 exhibited approximately 10-fold higher binding ability than BAT6026-wt to Fcγ-RIIIa 158V and 158F both variants.

**Table 1:** Kinetic parameters of BAT6026 and BAT6026-wt binding to Fcγ-RIIIa 158V or Fcγ-RIIIa 158F.

Sample	FcγRIIIa 158F			FcγRIIIa 158V		
	k_on_(1/Ms)	k_dis_(1/s)	k_D_ ^a^ (M)	k_on_(1/Ms)	k_dis_(1/s)	k_D_ ^a^ (M)
BAT6026	3.37E+05	6.32E-02	1.88E-07	3.30E+05	1.87E-02	5.67E-08
BAT6026-wt^b^	4.52E+04	1.13E-01	2.51E-06	2.17E+05	1.05E-01	4.81E-07

FcgR, gamma Fc receptor; k_on_, association rate; k_dis_, dissociation rate; k_D_, equilibrium dissociation constant.

a K_D_ values are calculated from the ratio of the kinetic constants as K_D_ = k_dis_/k_on_.

b BAT6026-wt, the BAT6026 Fc variant with wilde type IgG1 Fc.

**Figure 3:**
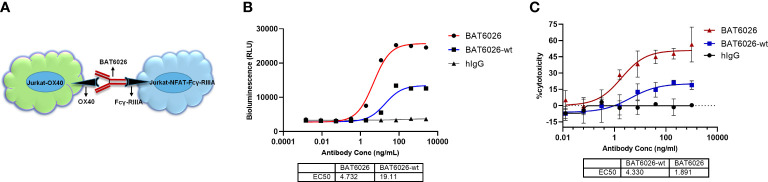
Enhanced ADCC activity of BAT6026 examined using cell-based function assays. **(A)** Schematic diagram of the ADCC Reporter Bioassay based on NFAT luciferase reporter assay. Jurkat-OX40 cells (target) were co-incubated with Jurkat–NFAT-FcγRIIIa reporter cells (effector) at an effector-to-target cell ratio of 2.5:1 in the presence of various concentrations of BAT6026 or BAT6026-wt. After incubation for 4 hours at 37°C, luciferase activity was determined. **(B)** ADCC activity of BAT6026 and BAT6026-wt were examined using the NFAT luciferase reporter assay. Human IgG (hIgG) was nonspecific IgG used as a negative control. **(C)** PBMCs from healthy donor, as effector cell, were co-incubated with Jurkat-OX40 cells (target cell) at an effector-to-target cell ratio of 25:1 in the presence of various concentrations of BAT6026 or BAT6026-wt. After incubation for 4 hours at 37°C, the released LDH was determined.

A cell-based functional assay, ADCC Reporter Bioassay system, was developed to measure the ADCC activity of BT6026 (**Figure 3A**). In this assay, the target cell was Jurkat cell stably overexpressing OX40, and the effector cell was Jurkat cell stably overexpressing Fcγ-RIIIa and a luciferase reporter driven by an NFAT response element (32). Following OX40 antibody binding to OX40 on the target cell and crosslinked by Fcγ-RIIIa on the effector cell surface, the signal pathway downstream of Fcγ-RIIIa was activated and culminated in activation of the luciferase reporter gene (**Figure 3A**) (33). As shown in **Figure 3B**, BAT6026 exhibited an EC50 value of 4.73 ng/ml, while BAT6026-wt exhibited an EC50 value of 19.11 ng/ml, indicating the ADCC activity of BAT6026 was enhanced approximately 4-fold. Human IgG (hIgG) was nonspecific IgG, which did not bind to OX40-expressing cells and was used as a negative control.

Another cell-based assay was applied to detect the *in vitro* ADCC activity of BT6026. PBMCs from healthy human as the effector cell were incubated with anti-OX40 antibody and the target cell, Jurkat cell expressing OX40. The effector cells would directly kill the target cells upon activated by antibody-mediated FcγRs clustering (33). BAT6026 showed obviously stronger ADCC activity than BAT6026-wt in this assay (**Figure 3C**), similar to the results of another assay (**Figure 3B**).

### Anti-tumor effect of BAT6026 in humanized syngeneic mice tumor model

**Figure 4:**
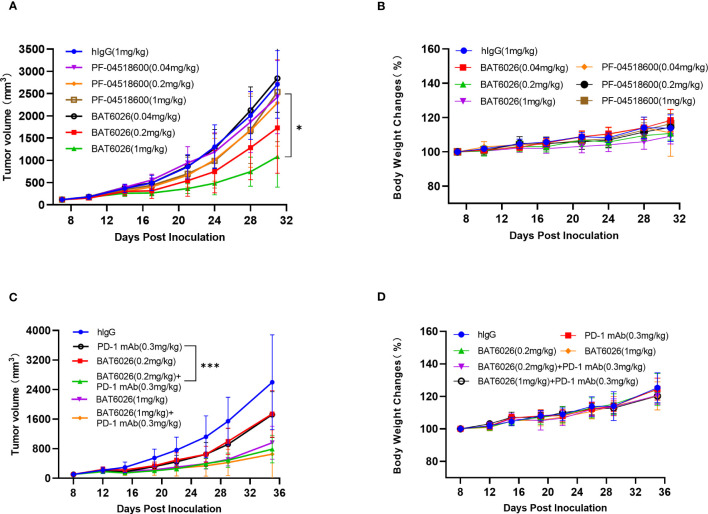
BAT6026 showed significant efficacy alone and in combination with anti-PD1 in mouse MC38 tumor model. **(A)** MC38 murine colon carcinoma cells were subcutaneously inoculated in OX40-humanized mice. When tumor reaches a mean volume of 119 mm^3^ at day 8 post tumor inoculation, animals were grouped (n=8) and dosed with hIgG control (1mg/kg), BAT6026 (1, 0.2, and 0.04mg/kg) or PF-04518600 (1, 0.2, and 0.04mg/kg) by intraperitoneal injection once every three days for a total of 6 times. **(B)** The body weight changes of the mice were measured twice a week and recorded after dosing. **(C)** MC38 cells were subcutaneously inoculated in OX40/PD1-dual-humanized mice. When tumor reaches about 100 mm^3^ at day 8 post tumor inoculation, hIgG control (1mg/kg), BAT6026 (0.2mg/kg), PD-1 antibody (BAT1308) (0.3mg/kg) or the combination dose was administered to the mice (n=8) by intraperitoneal injection once every three days for a total of 6 times. **(D)** The body weight changes of the mice were measured twice a week and recorded after dosing. All data was shown as mean ± SEM. *p < 0.05, ***p < 0.001.

BAT6026 did not bind to murine OX40, thus OX40-humanized mice were used to study the effect on tumor growth. OX40-humanized mice were generated by replacing the ECD of mouse OX40 with that of human OX40 in mouse blastocyst. After establishing the homozygous humanized OX40 mice, the expression of humanized OX40 in T cells upon activation has been confirmed (data not shown). As shown in **Figure 4A**, BAT6026 demonstrated a dose-dependent efficacy in inhibiting syngeneic MC38 mouse tumor growth. The mean tumor volume of BAT6026 in the 1 and 0.2 mg/kg groups at 31 days post-tumor inoculation showed a statistically significant difference compared with that of the isotype control group. The relative tumor growth inhibition (TGI) of 1 and 0.2 mg/kg BAT6026 groups was 62.8% and 37.7%, respectively. In contrast, the relative TGI of the same dose groups of PF-04518600 were merely 6.6% and 16.1%, respectively. These data demonstrated that the efficacy of BAT6026 on tumor growth inhibition was significantly stronger than that of PF-04518600.

To enhance T cells activation, combination treatment with PD-1, PD-L1, or CTLA-4 antibodies is a feasible approach for anti-OX40 immunotherapy. Therefore we examined the effect of combination treatment of BAT6026 with an anti-PD1 antibody, BAT1308, in a syngeneic MC38 mouse tumor model. Since BAT1308 did not bind to murine antigen either, PD-1/OX40-dual-humanized mice were used for the study. As shown in **Figure 4C**, combination treatment of 0.2 mg/kg BAT6026 plus 0.3 mg/kg BAT1308 resulted in a statistically significant difference in the mean tumor volume compared to that caused by each monotherapy (p=0.0025 for BAT6026 and p=0.0027 for BAT1308) at 35 days post-tumor inoculation (**Supplementary Table 2**). The relative TGI of 0.2 mg/kg BAT6026 group and 0.3 mg/kg BAT1308 group were 35.2% and 34.4%, respectively, while that of the combination treatment group was 72.7%. A similar trend of efficacy enhancement was also observed in the combination treatment of 1 mg/kg BAT6026 plus 0.3 mg/kg BAT1308 compared to BAT6026 monotherapy. However the enhancement was not as apparent as with 0.2 mg/kg BAT6026 dosing, likely due to the already high TGI (65.7%) of 1 mg/kg BAT6026 monotherapy (**Figure 4C** and **Supplementary Table 2**). Together, these results showed that combination treatment of BAT6026 and BAT1308 was significantly more effective than monotherapy on tumor growth inhibition.

Safety evaluation in the mouse tumor studies was based on animal death and body weight changes (**Figures 4B, D**). All treatment groups showed no deaths or signs of serious toxicity and the drug was well tolerated throughout the treatment (data not shown).

### BAT6026 reduced Tregs proportion and increases CD8+ T cells proportion in tumors​

To further explore the pharmacodynamic effects of BAT6026 and its mechanistic difference from BAT6026-wt in tumor, we analyzed the proportion and activation status of tumor-infiltrated T cells in mouse tumors treated with these two OX40 antibodies. Because human IgG1 is equivalent to mouse IgG2a in terms of interaction between FC domain and FC receptors (34, 35), we replaced the FC domain of BAT6026s with the mouse IgG2a FC domain to form two hybrid antibodies, BAT6026-mIgG2a-wt (normal ADCC) and BAT6026-mIgG2a (enhanced ADCC), which was completely devoid of fucose modification (**Supplementary Table 3**). OX40-humanized mice inoculated with MC38 tumors were treated with BAT6026-mIgG2a, BAT6026-mIgG2a-wt or vehicle at days 9 and 14 post-tumor inoculation. Tumors and spleens were collected and analyzed using flow cytometry on day 15.

**Figure 5:**
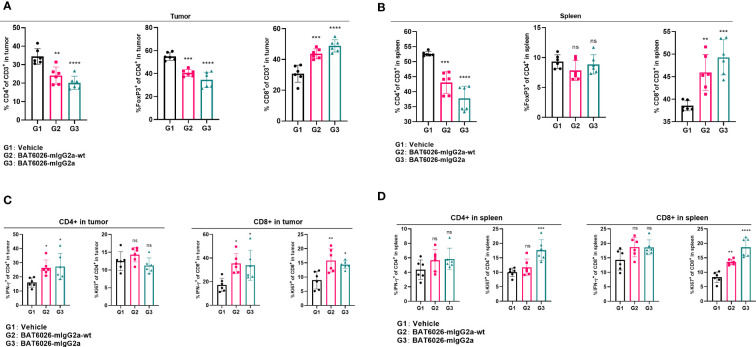
BAT6026 treatment decreased Tregs proportion, increased CD8+ T cell proportion and activated both CD4+ and CD8+ T cells in tumor. OX40-humanized mice bearing MC38 tumor were dosed with 5 mg/kg BAT6026-mIgG2a or BAT6026-mIgG2a-wt on days 9 and 14 post tumor injection. Single cell suspensions from tumors and spleens were collected and analyzed by flow cytometry 48 hrs after the last dose. **(A)** The percentage of CD4+ T cells, Treg and CD8+ T cells in tumor. **(B)** The percentage of CD4+ T cells, Treg and CD8+ T cells in spleen. **(C)** The percentage of IFN-γ+ or Ki67+ CD4+ and IFN-γ+ or Ki67+ CD8+ T in tumor. **(D)** The percentage of IFN-γ+ or Ki67+ CD4+ and IFN-γ+ or Ki67+ CD8+ T cells in spleen. Data shown as mean ± SEM, N=6. Ordinary one-way ANOVA was used (*p < 0.05, **p < 0.01, ***p < 0.001, ****p < 0.0001. ns, no significant difference).

As shown in **Figure 5A**, the proportions of CD4+ T cells and Treg cells in tumors of the BAT6026-mIgG2a-wt group and BAT6026-mIgG2a group were significantly reduced than those of the vehicle control group. This effect was more pronounced in the BAT6026-mIgG2a group, likely because of the stronger ADCC effect. Meanwhile, the proportion of intra-tumoral CD8+ T cells was significantly increased in BAT6026-mIgG2a-wt and BAT6026-mIgG2a groups, with the latter group being even more apparent (**Figure 5A**). These data suggest that ADCC-enhanced BAT6026-mIgG2a may deplete more intra-tumoral Treg cells than BAT6026-mIgG2a-wt, thus resulting in more CD8+ T cells infiltration inside the tumor. In spleen, the same trend of effect on CD4+ and CD8+ T cells caused by treatment with these two OX40 antibodies was detected. However, unlike in tumors, the proportion of Tregs in spleen did not change significantly after OX40 antibodies treatment (**Figures 5B**).

The activation status of T cells in mice treated with these OX40 antibodies were also examined. Compared with the control group, the percentage of IFN-γ+ CD4+ or IFN-γ+ CD8+ T cells was significantly increased in tumor after treatment with either OX40 antibody, and the level of increase was similar between these two antibodies (**Figures 5C**). These data suggest that treatment with the two OX40 antibodies may cause different levels of decrease on CD4+ T cells and increase on CD8+ T cells in tumor (**Figures 5A**), yet they cause a similar level of IFN-γ expression, implicating T cell activation, on CD4+ and CD8+ cells, in tumor. Roughly similar phenomena were observed in the spleen following treatment with these OX40 antibodies (**Figures 5B, D**). Furthermore, the proportion of proliferating tumor-infiltrated CD8+ T cells (Ki67+), but not CD4+ T cells, was significantly increased in tumor after administration of either OX40 antibodies (**Figures 5C**).

### Safety profile of BAT6026 in cynomolgus monkeys

Prior to clinical trial in human, a GLP toxicology study of BAT6026 was performed in cynomolgus monkeys. In a repeat-dose toxicology study, 40 cynomolgus monkeys (20 females and 20 males) were administered BAT6026 (1, 5 or 30 mg/kg) *via* intravenous infusion once a week for a total of 5 doses, then the animals were allowed for a 4-week recovery phase. The results showed that repeated infusions of BAT6026 to monkeys were well tolerated at these doses, with main changes observed of decreased neutrophils, transient increased IL-6 and slight histological changes in spleen/liver (**Supplementary Table 4**). Therefore, the highest non-severely toxic dose (HNSTD) in the study was determined to be 30 mg/kg.

## Discussion

As a novel and fully human IgG1 monoclonal antibody, BAT6026 was obtained through screening a yeast display library and affinity maturation process. BAT6026 demonstrated a high and specific affinity to purified antigen and cell-surface human OX40. To strengthen one of its modes of action on Treg depletion, BAT6026 was expressed as an ADCC-enhanced antibody, which differentiates it from other anti-OX40 antibodies for cancer indication. Compared with current anti-OX40 field leader, PF-04518600, BAT6026 demonstrated superior activities on binding to OX40, blocking binding of OX40L to OX40, activation of T cells and SEB-pretreated PBMCs, as well as tumor inhibition in MC38 tumor model of OX40-humanized mice. BAT6026 also showed a significantly synergistic effect on tumor inhibition when combined with an anti-PD-1 antibody. We further investigated the effect of ADCC enhancement of BAT6026 on the proportions of CD4+ T cells and Tregs in mouse tumor, and the results showed that these cells were significantly reduced in mice treated with BAT6026 compared to its cognate antibody with regular ADCC, BAT6026-wt.

It has been reported that the expression level of OX40 on Tregs is much higher than that on CD8+T cells in many types of tumors (17–19), suggesting the tumor inhibition mechanism of OX40 antibody may be predominantly mediated *via* Treg suppression. Indeed, Bulliard et al. demonstrated that anti-OX40 antibody treatment caused mice Colon26 tumor regression and concomitant elimination of intratumoral Treg cells *via* FcγRs-mediated ADCC effect (15). We used a pair of OX40 antibodies with approximately four-fold difference in their ADCC activity, and found that treatment with ADCC-enhanced BAT6026 resulted in significantly fewer CD4+ T cells and Treg cells in mice tumor than with BAT6026-wt. The *in vivo* efficacy of these two antibodies was also compared in an MC38 syngeneic mouse tumor model. Although not with statistically significant difference, BAT6026 showed a trend of stronger anti-tumor effect in the OX40-humanized mice model than BAT6026-wt (TGI 38.3% versus 21.1%) (data not shown). The reason that difference in TGI was not large could be because both antibodies were human IgG1 and thus in mouse model the ADCC activity difference was reduced (34). Afterall, our data were generally consistent with the report of Bulliard et al. and support the notion that the predominant anti-tumor mechanism of OX40 antibody is through Treg suppression.

In **Figure 5A**, we observed that Treg in mouse tumor was reduced when treated with BAT6026, but not completely depleted. This may be due to the fact that some Treg cells express little or no OX40 inside the tumor. Moreover, the number of effector cells, such as NK cells, inside the tumor is limited, which may lead to limited depletion of Treg *via* ADCC effect. Treg depletion/reduction was not observed in spleen (**Figure 5B**). Since Treg cells in spleen may be inactive, the expression level of OX40 on these cells is none or very low, which may prevent Treg depletion/reduction.

BAT6026 demonstrated a dose-dependent and powerful efficacy in inhibiting MC38 tumor growth in syngeneic mice, with a stronger efficacy than PF-04518600 and BAT6026-wt. Furthermore, in an OX40/PD1 dual humanized mouse tumor model, combination treatment of BAT6026 and anti-PD1 BAT1308 was significantly more effective than single treatment. A manuscript by Messenheimer et al. reported that concurrent administration of PD-1 and OX40 antibodies could suppress the therapeutic effects of OX40 antibody in an orthotopically transplanted MMTV-PyMT mammary tumor model. However, sequential combination treatment of OX40 antibody followed by PD-1 antibody (but not the reverse order) resulted in significant increases in therapeutic efficacy (36). Another research found that addition of PD-1 antibody exhibited a detrimental effect on the antitumor response of OX40 antibody when they were concurrently administered in TC-1 tumor model (37). So far these are the only two manuscripts describing such attenuation effect on tumor inhibition by combination treatment. Many other preclinical studies in the field have reported that simultaneous administration of OX40 and PD-1 antibodies resulted in a significant synergistic antitumor effect (5, 38), which is consistent with our report here. These different results could be due to the different mouse tumor models used. To clarify the consequences of different administration orders for combination therapy of OX40 and PD-1 antibodies, more in-depth researches are required.

BAT6026 was expressed as an antibody completely devoid of fucose modification on N297 of Fc domain. Nonfucosylated modification can increase the affinity of Fc domain to FcγRIIIa 5-10 folds or even more (29, 39). Besides enhancing ADCC effect through stronger binding to NK cell, the increased Fc-FcγRIIIa interaction has recently been reported to promote the communication between APC and T cells bound with immune checkpoint antibodies, which further activated T cells and strengthened their tumoricidal activity (40). Thus, nonfucosylated BAT6026 can enhance the activation of CD4+ and CD8+ T cells, as well as depletion of Tregs. Compared to PF-04518600, another advantage BAT6026 holds is its ability to induce cross-linking provided by FcγRs. OX40 is a member of TNFRSF and its activation requires aggregation induced by binding to cell surface trivalent ligand OX40L, or to antibodies and subsequently cross-linked by FcγRs (13). BAT6026 is an ADCC-enhanced IgG1 antibody, while PF-04518600 is a regular IgG2 antibody, which has lower affinity to FcγRs (41), and thus weaker ability to induce cross-linking. In the T cell activation experiment, PF-04518600 exhibited a notably lower level of maximal activation signal than BAT6026 (**Figure 2B**). This difference could be due to its weaker ability to induce cross-linking provided by FcγRs. Recently, two articles reported that the reduced affinity of costimulatory receptors (CD40/4-1BB/OX40) antibodies let them achieve superior agonism by Fc receptors-independent clustering (42, 43). In our hands, we have also observed a similar phenomenon on OX40 antibodies. OX40 antibodies with relatively low affinity (10-20nM), not high affinity (<1nM), can activate OX40 in the absence of Fc receptors. However, when Fc receptors are present, OX40 antibodies with high affinity can activate OX40 at similar level as low-affinity antibodies (data not shown). After all, leaning toward more effectively depleting OX40+ Treg in tumors, BAT6026 with high affinity was chosen as the clinical candidate.

BAT6026 did not show significant side effects in preclinical toxicology study. Also, most OX40 antibodies did not show significant side effects in clinical trials. This may be due to the fact that OX40 is inducible and mainly expressed within 24-72 hours after activation of CD4+ and CD8+T cells, but not on resting T cells. BAT6026 is currently in phase I clinical trial administered alone (NCT05105971) and in combination with PD-1 antibody (NCT05109650). With these superior characteristics and a favorable safety profile in preclinical GLP toxicity research, we look forward to exposing the clinical superiority of BAT6026.

## Data availability statement

The original contributions presented in the study are included in the article/**Supplementary Material**. Further inquiries can be directed to the corresponding author.

## Ethics statement

The animal study was reviewed and approved by The Institutional Animal Care and Use Committee of Biocytogen Pharmaceuticals (Beijing) Co., Ltd.

## Author contributions

ShL, DZ, XL, XM, CZ, CX, HY carried out the experiments. ShL, CQ, JL, CL, SfL, J-CY conceived of the study and participated in its design and coordination. ShL and J-CY drafted the manuscript. All authors read and approved the final manuscript.
