# Supplementary material for: BAT6026, a novel anti-OX40 antibody with enhanced antibody dependent cellular cytotoxicity effect for cancer immunotherapy
Source: Front Oncol. 2023 Jul 28;13:1211759. doi: 10.3389/fonc.2023.1211759 (PMC10421724; doi:10.3389/fonc.2023.1211759)
Supplement: Supplementary file 1 [file DataSheet_1.docx]

**Supplement Figures：**

**Figure.S1**

A，

B，

C，

D，

Figure. S1. Effect of BAT6026 on the release of cytokines from unactivated human PBMCs. Unactivated PBMCs was incubated with various concentrations of BAT6026 for 4 days, and the secreted IL-2(A), INF-γ(B), TNF-α(C) and IL-6(D) were measured by ELISA.

**Supplement Tables：**

**Supplementary Table 1** Glycosylation of BAT6026 and nonfucosylated BAT6026-wt

|  | G0-GN | G0F-GN | G0 | G0F | Man5 | Man6 | Man8 | G1 | G1' | G1F | G1F' | G2 | G2F | others |
| --- | --- | --- | --- | --- | --- | --- | --- | --- | --- | --- | --- | --- | --- | --- |
| BAT6026 | 3 | 0 | 66.29 | 0 | 1.22 | 0.13 | 0.34 | 8.73 | 5.33 | 0 | 0 | 0.75 | 0 | 17.21 |
| BAT6026-wt | 0.28 | 1.08 | 1.32 | 68.56 | 0.87 | 0 | 0 | 0.58 | 0.17 | 15.25 | 6.52 | 0 | 1.68 | 3.69 |

**Supplementary Table 2** Tumor inhibition activity of BAT6026 and BAT1308 combination treatment in OX40/PD1-dual-humanized mice tumor model

| Group | Mean Tumor Volume (mm3) | Tumor volume P‑value (compared with hIgG ) | TGI(%) | P-value (compared with BAT1308 0.3mg/kg) | P-value (compared with BAT6026 0.2mg/kg) |
| --- | --- | --- | --- | --- | --- |
| hIgG Control | 2600±455 | -- | -- | -- | -- |
| BAT1308 0.3 mg/kg | 1721±220 | 0.104 | 35.2 | -- | -- |
| BAT6026 1 mg/kg | 962±158 | 0.004 | 65.7 | -- | -- |
| BAT6026 0.2 mg/kg | 1741±223 | 0.112 | 34.4 | -- | -- |
| BAT6026 1 mg/kg + BAT1308 0.3 mg/kg | 653±232 | 0.002 | 78.0 | 0.0048 | -- |
| BAT6026 0.2 mg/kg + BAT1308 0.3 mg/kg | 787±132 | 0.002 | 72.7 | 0.0027 | 0.0025 |

**Supplementary Table 3** Glycosylation of nonfucosylated BAT6026-mIgG2a

|  | G0-GN | G0 | G1-GN | G1-GN` | Man5 | G1 | G1' | G2-GN | G2 | G1S1 | Man7 | G2S2 | Man9 | other |
| --- | --- | --- | --- | --- | --- | --- | --- | --- | --- | --- | --- | --- | --- | --- |
| BAT6026-mIgG2a | 0.82 | 31.19 | 0.19 | 0.43 | 0.65 | 41.84 | 5.61 | 1.40 | 12.67 | 0.16 | 0.57 | 0.10 | 0.22 | 4.14 |

**Supplementary Table 4** Repeat-dose toxicology Study Summary of BAT6026 in Cynomolgus Monkeys.

| Study Title | Conclusion |
| --- | --- |
| Hematology | Some males at ≥5 mg/kg and some females at 5 mg/kg were found with decreased neutrophil on Day 16 and/or Day 30. |
| Cytokines | Compared with the animals in vehicle control group and/or pre-dose individuals, some females in dose groups were observed with transient increased IL-6 at 12 h or 24 h after the first dosing. |
| Histopathology | At the end of dosing phase, minimal decreased cellularity of lymphocytes in spleen and minimal or mild hypertrophy of kupffer cells were present in some males at ≥ 5 mg/kg. Minimal or mild increased cellularity of neutrophils in spleen, minimal increased kupffer cell in liver and mild hypertrophy of cortex in adrenal gland were also present in monkey dosed with 30 mg/kg of BAT6026. |
| Others | Neither the females nor the males at any dose were observed with test article-related changes in clinical observations, ophthalmology, body weight, food consumption, hematology, coagulation, plasma chemistry, immune globulin, lymphocyte immunophenotype, urine, gross pathology, organ weights, local irritation at administration site, cardiovascular, respiratory or central nerve systems. |
